# Caffeine supplementation improved movement patterns and reactive agility in rugby sevens matches in male collegiate players

**DOI:** 10.1080/15502783.2024.2441763

**Published:** 2024-12-16

**Authors:** Chang-Li Hsueh, Cheng-Yen Sun, Chen-Kang Chang

**Affiliations:** a National Taiwan University of Sport, Department of Sport Performance, Taichung, Taiwan; b Chang Jung Christian University, Department of Athletic Sports, Tainan, Taiwan

**Keywords:** Acceleration, deceleration, global positioning system, reactive agility, repeated high-intensity effort

## Abstract

**Purpose:**

Rugby sevens is a high-intensity contact sport often played in two-day tournaments. Caffeine is widely used by rugby players for its performance-enhancing effects. This study aimed to investigate the impact of caffeine supplementation on various performance metrics, including distance covered at different speeds, acceleration, deceleration, collisions, and repeated high-intensity efforts across four matches over two consecutive days in collegiate male rugby sevens players. Reactive agility, a key performance attribute in rugby sevens, was also assessed before each match.

**Methods:**

A position-matched, double-blind, randomized crossover design was employed, with six male collegiate rugby players (mean height: 1.78 ± 0.09 m, mean weight: 81.3 ± 9.2 kg, mean age: 21.5 ± 0.8 years) participating in two trials. Each trial consisted of a two-day tournament, with two matches per day. Performance was monitored using global positioning system units to track distance covered in various speed zones, as well as total distance, frequency of acceleration, deceleration, collisions, and repeated high-intensity efforts.

**Results:**

The results indicated that in the placebo trial, participants covered significantly more distance at a walking pace (0–6 km/h) in match 4 compared to match 3 (match 3: 480.3 ± 32.7 m; match 4: 629.4 ± 21.3 m, *p* < 0.001, d = 0.117). In the caffeine trial, players covered significantly more distance at a jogging pace (6–12 km/h) in match 4 compared to the placebo trial (caffeine: 405.9 ± 9.8 m; placebo: 303.6 ± 20.2 m, *p* = 0.015, d = 1.693). Reactive agility was significantly better in the caffeine trial before match 3 (caffeine trial: 1.80 ± 0.17 s; placebo trial: 2.07 ± 0.18 s, *p* = 0.038, d = 0.858).

**Conclusions:**

Caffeine supplementation at 3 mg/kg may increase jogging and reduce walking and standing in the final match of a two-day rugby sevens tournament, while also improving reactive agility on the second day. This suggests that by mitigating fatigue in the later stages of the tournament, caffeine allowed players to shift from low-intensity activities to higher-intensity efforts. These adjustments may improve both offensive and defensive performance during rugby sevens matches. Therefore, rugby sevens players could benefit from taking caffeine supplements in the later stages of 2-day tournaments to optimize their performance.

## 1. Introduction

Rugby sevens is a contact team sport characterized by its intermittent nature, featuring alternating periods of high-intensity activities such as sprinting, acceleration, deceleration and physical collisions, alongside low-intensity activities like standing, walking, and jogging [1]. During matches, players cover approximately 1.6 km and maintain a playing intensity exceeding 80% of their maximal heart rate as they strive to gain or defend field position [2]. Since its formal inclusion in the Olympic Games in 2016, interest in the physical and technical performances of rugby sevens players has increased significantly [3–5]. The sport is typically played in a two-day tournament format with two to three matches per day. Consequently, nutrition interventions aimed at enhancing performance and facilitating recovery are critical for success in rugby sevens competitions.

Caffeine is a popular supplement among professional and amateur rugby players due to its ability to enhance various aspects of exercise performance [6]. It has been shown that supplementing with 3–6 mg/kg of caffeine significantly increases average power output and improves time trial performance in endurance exercise [7]. Another meta-analysis revealed consuming 3–6 mg/kg caffeine before exercise significantly enhances average and peak power output in the Wingate test, demonstrating caffeine’s ability to improve anaerobic performance [8]. Additionally, caffeine doses ranging from 3 to 7 mg/kg significantly enhance maximal muscle strength and power [9]. In team sports characterized by high-intensity intermittent activity, 3–6 mg/kg caffeine supplementations significantly enhance single and repeated sprint velocity, total distance covered, distance covered at sprint velocity, and the number of sprints performed during competitions [10]. However, most studies focus on single match on a single day.

A previous study has shown that caffeine supplementation can increase distance covered at different speed zones in three consecutive rugby sevens matches in a single day among elite female players [11]. However, there is currently a lack of studies investigating these effects in male rugby sevens players. Two review articles suggested that the effect of caffeine on team sport performance may differ between sexes [10,12]. For example, caffeine supplementation significantly improved repeated sprint performance during competitions in male [10] but not in female players [12]. Moreover, given that rugby sevens tournaments typically span two days, it remains unclear whether caffeine supplementation before each match can be effective on the second day of competition. The purpose of this study was to investigate the effects of caffeine supplementation on performance variables, including distance covered at different speed zones, acceleration and deceleration, collisions, and repeated high-intensity effort, across four matches over two consecutive days in collegiate male rugby sevens players. Reactive agility, a crucial ability in rugby sevens performance, was also assessed before each match. It is hypothesized that caffeine supplementation can improve match performance and reactive agility.

## 2. Methods

### 2.1. Study design

**Figure 1.**
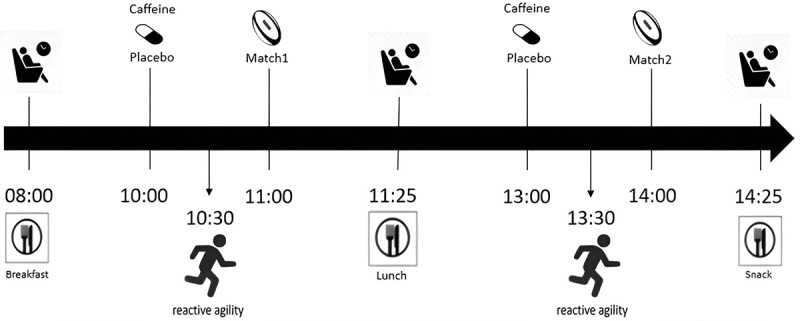
Study protocol.

This study employed a position-matched, double-blind, randomized crossover design. Pairs of participants with similar playing positions, specifically forwards or backs/scrum halves, were randomly assigned to either the caffeine or placebo trial. Each trial comprised a two-day rugby sevens tournament, with participants playing two matches per day. The study design is illustrated in Figure 1. Following a 13-day washout period, participants switched to the alternate trial. Both the participants and the research personnel were blinded to the supplementation condition throughout the study period, with the assignment of supplements disclosed to research personnel only after all data analyses were completed. The study protocol was approved by the Human Research Ethics Committee of Jen-Ai Hospital, Taichung, Taiwan (111–03). Written informed consent was obtained from all participants, and all experiments were conducted in accordance with relevant guidelines and regulations. The protocol is registered in ClinicalTrials.gov (NCT06612463).

### 2.2. Participants

Eight male rugby players from a Division I university team were recruited for this study. All participants had undergone regular rugby training for at least three years. The exclusion criteria included musculoskeletal injury within one month prior to the study or the presence of major cardiovascular or metabolic diseases. During the second match of the first phase, one back player sustained an injury and was replaced by another back player who completed the remaining matches in both phases of the study. However, only data from the six participants who played all eight matches were included in the analysis. These six participants – comprising three forwards and three backs/scrum halves – had a mean height of 1.78 ± 0.09 m, a mean weight of 81.3 ± 9.2 kg, and a mean age of 21.5 ± 0.8 years. The sample size was limited because of the number of players in a rugby sevens match and all participants had to participated in four matches.

### 2.3. Rugby sevens match

On both the first and second day of each trial, participants competed in a seven-a-side rugby match at 11:00 and 14:00 against the same opponent, another Division I university team. Each match consisted of two seven-minute halves with a one-minute half-time. Qualified referees oversaw the matches in accordance with official regulations. Player substitutions were permitted for the opposing team but not for the participants of the study.

### 2.4. Supplementation

In the caffeine trial, participants ingested a capsule containing 3 mg/kg caffeine (Wako Pure Chemical Industries, Ltd., Osaka, Japan) 60 min prior to the match. In the placebo trial, participants ingested a capsule containing starch (Chung-Yu Biotech Co LTD, Taichung, Taiwan) at the same time point. The supplements prior to the first match were consumed approximately 100 min after breakfast. The supplements prior to the second match were consumed approximately 75 min after lunch. The capsules used in both trials were identical in appearance. The caffeine and placebo supplements were prepared by a laboratory technician who was independent of the study.

### 2.5. Dietary intervention

On the day before each trial, participants were provided with breakfast, lunch, and dinner purchased from local diners. The diet supplied 5.5 ± 0.2 g/kg of carbohydrates, 1.9 ± 0.2 g/kg of protein, 1.1 ± 0.1 g/kg of fat, and 38.2 ± 0.7 kcal/kg of energy. On match days, participants consumed breakfast at 08:00, lunch after the first match, a snack after the second match, and dinner at 17:30 (Figure 1). The nutritional content of each meal is detailed in Table 1. Participants consumed the same food in both trials and were allowed to drink water ad libitum throughout the trials. The participants were instructed to avoid consuming any caffeine-containing food on the day before and during the 2-day trials.

**Table 1.** Dietary composition of meals on match days.

	Carbohydrate (g/kg)	Protein (g/kg)	Fat (g/kg)	Energy (kcal/kg)
Breakfast	0.7 ± 0.1	0.2 ± 0.0	0.2 ± 0.0	5.3 ± 0.7
Ham and cheese sandwich				
Soy milk				
Lunch	1.7 ± 0.2	0.1 ± 0.0	0.1 ± 0.0	8.1 ± 1.0
Rice ball				
Cereal bar				
Banana				
Energy jelly				
Sport drink				
Snack and dinner	3.8 ± 0.6	1.1 ± 0.1	0.7 ± 0.0	25.4 ± 2.8
Ham and cheese sandwich (after match)				
Meal box				
Boiled chicken breast				
Soy milk				

### 2.6. Reactive agility test

A reactive agility test was conducted approximately 30 min before each match following a proper warm-up. The warm-up procedure was consistent across all tests. The participants sprinted with the ball, passing through a light gate (WITTY-SEM, Microgate, Bolzano, Italy), after which an indicator light immediately displayed a left or right direction. The participants had to respond by changing direction according to the indicator and continue moving until they passed the second light gate positioned 1.5 m away from the indicator [13]. The fastest time from two attempts was used for analysis.

### 2.7. Global positioning system

Participants wore an appropriately sized vest housing the portable global positioning system unit (Vector S7, Catapult, Melbourne, Australia) between the scapulae, with the same unit used for each match. The device sampled at a rate of 1000 hz and was integrated with a triaxial accelerometer sampling at 100 hz. After the match, the devices were connected to a computer. Using specialized software (Openfield, version 3.9.0, Catapult), the data was downloaded, excluding the periods of pre-match warm-up and halftime. The following parameters were analyzed.

#### 2.7.1. Total distance and distance covered in speed zones

Movement speed must be maintained for at least 1 s, and the interval between two movements must exceed 1 s. If the time interval between two movements is less than 1 s, it is considered part of the previous movement and analyzed as a single movement. A speed record is terminated when the speed drops below the designated speed range for more than 1 s [14]. Speed is divided into six zones: standing and walking (0–6 km/h), jogging (6–12 km/h), cruising (12–14 km/h), striding (14–18 km/h), high-intensity running (18–20 km/h), and sprinting (>20 km/h) [3,11]. The distance covered in each speed zone and the total distance covered were recorded.

#### 2.7.2. Acceleration and deceleration

For an activity to be included in the analysis, the movement speed must exceed 5 km/h, and the acceleration must be greater than 2 m/s^2^ or the deceleration must be less than −2 m/s^2^, sustained for more than 0.9 s. If the interval between two movements is less than 1 s, the subsequent movement will be combined with the previous one for analysis [14]. The frequency and distance covered during these acceleration and deceleration phases were recorded.

#### 2.7.3. Collision

Collision is defined as the number of times a participant accumulates 0.5 s standing after body contact resulting in a fall. Specific collision events include rucks, tackles, and instances where a player falls after contact [15].

#### 2.7.4. Repeated high-intensity effort

A repeated high-intensity effort is defined as the completion of three or more high-intensity actions in succession, with rest periods of no more than 21 s between each action. The criteria for high-intensity actions include a movement speed of at least 14 km/h, an acceleration of at least 2 m/s^2^, and a deceleration of at least −2 m/s^2^ [1]. The rest period is defined as a duration without any high-intensity action.

### 2.8. Statistical analysis

The results were presented as mean ± standard deviation. Most variables (105 out of 112) were normally distributed according to the Shapiro – Wilk test. Reactive agility before each match and movement variables in each match were analyzed using linear mixed models to account for individual differences among participants. In these models, the fixed effects were trial and time, while the random effect accounted for individual participant variability. When a significant time effect was identified, differences among matches within the same trial were analyzed using one-way repeated-measures analysis of variance and a Bonferroni test was used for post hoc comparisons. In the event of a significant trial effect, a paired t-test was used to identify the difference between the trials at the same time point. Effect sizes for paired t-test are expressed as Cohen’s d and categorized as small (0.2 ≤ d < 0.5), moderate (0.5 ≤ d < 0.8), large (0.8 ≤ d < 1.2), and very large (1.2 ≤ d) [16]. Based on the sample size of 6, the effect size of 1.18 can be detected with the power of 0.8, as estimated using the software G*Power 3.1. Data were analyzed using SPSS for Windows, version 23.0 (IBM, Armonk, NY, USA). Statistical significance was set at *p* < 0.05.

## 3. Results

Total distance and distance covered in each speed zone across the four matches in both caffeine and placebo trials are detailed in Table 2. A significant time effect was observed for the distance covered at speeds of 0–6 km/h (*p* = 0.001). Post hoc analysis indicated that, during the placebo trial, the distance covered at 0–6 km/h was significantly greater in match 4 compared to match 3 (match 3: 480.3 ± 32.7 m; match 4: 629.4 ± 21.3 m, *p* < 0.001, d = 0.117). Conversely, no significant differences were found in distance covered at 0–6 km/h across the four matches in the caffeine trial. In addition, a significant trial effect was observed for the distance covered at 6–12 km/h (*p* < 0.001). The post hoc analysis revealed that in match 4, participants in the caffeine trial covered significantly longer distance at 6–12 km/h than in the placebo trial (caffeine: 405.9 ± 9.8 m; placebo: 303.6 ± 20.2 m, *p* = 0.015, d = 1.693).

**Table 2.** Total distance and distance covered at different speed zones across four matches in caffeine and placebo trials.

					*P* value		
	Match 1	Match 2	Match 3	Match 4	Trial	Time	Trial x Time
Total distance (m)					0.054	0.633	0.703
Caffeine	1485.8 ± 67.9	1379.8 ± 42.0	1394.7 ± 50.3	1502.5 ± 89.9			
Placebo	1346.9 ± 83.8	1373.9 ± 64.1	1312.9 ± 46.6	1359.2 ± 67.5			
Distance at 0–6 km/h (m)					0.743	0.001	0.507
Caffeine	561.8 ± 21.8	536.1 ± 24.3	511.6 ± 24.4	585.9 ± 21.8			
Placebo	561.6 ± 17.5	547.2 ± 29.6	480.3 ± 32.7	629.4 ± 21.3*			
Distance at 6–12 km/h (m)					<0.001	0.746	0.563
Caffeine	371.1 ± 20.6	382.9 ± 35.6	363.4 ± 19.0	405.9 ± 9.8^†^			
Placebo	307.4 ± 22.0	338.6 ± 20.5	318.6 ± 29.0	303.6 ± 20.2			
Distance at 12–14 km/h (m)					0.262	0.814	0.459
Caffeine	145.6 ± 5.8	161.7 ± 16.9	148.3 ± 16.6	161.8 ± 12.7			
Placebo	136.4 ± 17.7	135.8 ± 17.7	164.8 ± 31.1	120.0 ± 21.8			
Distance at 14–18 km/h (m)					0.799	0.847	0.922
Caffeine	215.7 ± 25.2	205.2 ± 19.3	203.3 ± 28.2	199.4 ± 38.2			
Placebo	203.7 ± 49.7	207.8 ± 26.5	217.6 ± 30.2	170.8 ± 35.2			
Distance at 18–20 km/h (m)					0.388	0.686	0.245
Caffeine	90.6 ± 25.1	46.3 ± 7.8	61.4 ± 9.8	76.5 ± 18.1			
Placebo	54.1 ± 10.6	66.2 ± 15.1	60.8 ± 4.6	58.7 ± 11.6			
Distance at >20 km/h (m)					0.778	0.585	0.550
Caffeine	100.0 ± 19.5	47.5 ± 5.1	106.4 ± 42.9	72.6 ± 14.7			
Placebo	83.5 ± 21.1	77.4 ± 25.0	70.6 ± 21.4	76.2 ± 21.4			

*p < 0.05, significantly different from Match 3 in the placebo trial; ^†^*p* < 0.05, significantly different from Match 4 in the placebo trial.

Frequency and distance covered during acceleration and deceleration, as well as the frequency of collisions and repeated high-intensity efforts, are shown in Table 3 for both caffeine and placebo trials. No significant effect was found in any of the variables.

**Table 3.** Acceleration, deceleration, collision, and high-intensity repeated effort across four matches in caffeine and placebo trials.

					*P* value		
	Match 1	Match 2	Match 3	Match 4	Trial	Time	Trial x Time
Acceleration (n)					0.332	0.164	0.988
Caffeine	14.33 ± 0.62	17.67 ± 1.65	15.67 ± 1.26	19.83 ± 3.72			
Placebo	13.50 ± 1.65	16.33 ± 3.03	14.50 ± 2.11	17.67 ± 3.05			
Distance in acceleration (m)					0.106	0.348	0.988
Caffeine	58.03 ± 3.76	65.31 ± 7.33	64.51 ± 5.59	76.24 ± 12.65			
Placebo	49.89 ± 5.45	57.13 ± 9.65	51.85 ± 9.54	63.45 ± 12.88			
Deceleration (n)					0.115	0.270	0.869
Caffeine	16.00 ± 1.63	19.50 ± 0.92	19.17 ± 2.09	19.83 ± 2.70			
Placebo	13.67 ± 3.25	18.83 ± 2.61	14.67 ± 1.41	16.83 ± 2.80			
Distance in deceleration (m)					0.373	0.360	0.985
Caffeine	21.50 ± 2.19	26.50 ± 2.14	22.67 ± 2.84	26.33 ± 3.72			
Placebo	19.67 ± 2.65	22.87 ± 3.60	20.48 ± 3.67	25.28 ± 5.04			
Collision (n)					0.332	0.824	0.430
Caffeine	4.0 ± 0.5	4.3 ± 1.0	3.3 ± 0.6	4.8 ± 1.2			
Placebo	3.3 ± 0.9	4.5 ± 1.0	3.8 ± 0.9	2.5 ± 0.9			
Repeated high-intensity effort (n)					0.391	0.746	0.880
Caffeine	9.0 ± 0.4	8.0 ± 0.7	8.2 ± 0.7	9.0 ± 0.8			
Placebo	7.8 ± 0.9	8.2 ± 0.9	7.7 ± 0.8	8.5 ± 1.1			

**Figure 2.**
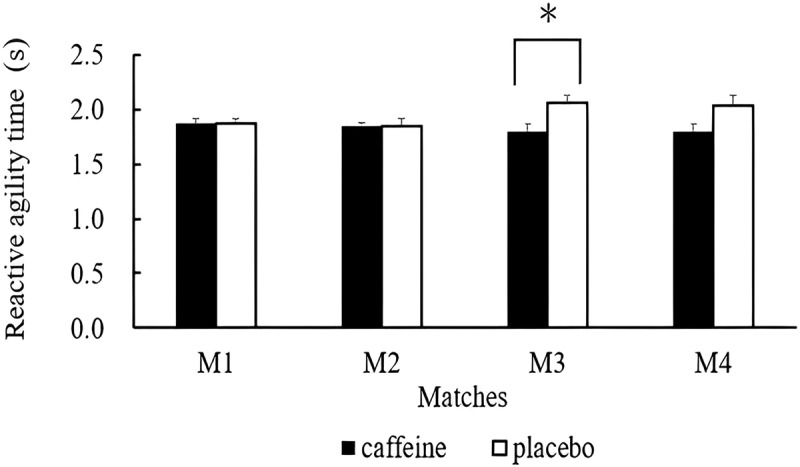
Reactive agility performance before each match in the caffeine and placebo trials.

Results from the reactive agility test conducted before each match are illustrated in Figure 2. A significant group effect (*p* = 0.008) was observed for reaction time. A trend of interaction effect (*p* = 0.054) was also observed. Post hoc analysis indicated that reactive agility performance was significantly better in the caffeine trial compared to the placebo trial before match 3 (caffeine trial: 1.80 ± 0.17 s; placebo trial: 2.07 ± 0.18 s, *p* = 0.038, d = 0.858).

*p > 0.05.

## 4. Discussion

This study suggested that caffeine supplementation 3 mg/kg before each match enhanced distance covered at 6–12 km/h while decreasing distance covered at 0–6 km/h in the last match of the two-day tournament in collegiate male rugby sevens players. Moreover, caffeine supplementation improved reactive agility before the first match on the second day of the tournament.

Increased jogging (6–12 km/h) distance at the expense of standing and walking (0–6 km/h) suggests that the participants in the caffeine trial maintained a higher level of activity to support both offensive and defensive plays in match 4. This heightened engagement can lead to quicker responses to game dynamics, better positioning, and more effective execution of strategies. Additionally, the reduced distance of low activity indicates lower fatigue levels among the participants in the caffeine trial. Previous research has indicated that caffeine supplementation can enhance performance across various team sports [10,17] due to its ability to cross the blood-brain barrier and acts as an antagonist to A1 and A2a adenosine receptors [18]. This action affects both the muscular and central nervous system. Caffeine reduces muscle fatigue, promotes the breakdown of triglycerides and glycogen, stimulates adrenaline release, enhances alertness, and improves mood [19]. In rugby sevens, the ergogenic effects of caffeine have been observed in elite female players. For instance, the consumption of an energy drink containing 3 mg/kg of caffeine significantly improved total distance and distance covered at speeds exceeding 12 km/h across three consecutive games [11]. Similarly, another study found that 3 mg/kg of caffeine enhanced the rate of body impacts during three consecutive international rugby sevens matches played on the same day [20]. The ergogenic effects of caffeine observed in elite female players differ from those in our study, where caffeine supplementation increased the distance covered at 6–12 km/h without affecting the number of collisions.

One potential reason for the differing effects is that the aforementioned studies pooled data from three matches, including both starters and substitutes from the same team, who inherently had varying playing times. This variation in playing time may have influenced the observed effects of caffeine supplementation, as starters, who generally play more minutes, could experience different physiological responses compared to substitutes, whose movement variables were significantly higher [21]. In contrast, the current study focused exclusively on participants who played the full duration of all four matches. This approach eliminates variability associated with different playing times and provides a more precise understanding of the potential impact of caffeine on rugby sevens performance.

Rugby sevens players experience substantial increases in plasma concentrations of H^+^ and lactate after matches, indicating acidosis and the high energy demands of the glycolytic pathway [22]. Additionally, intense running and collisions elevate plasma markers for muscle damage, such as creatine kinase [23]. Despite this physical strain, elite male players managed to maintain total distance and distance covered at various speed zones across three matches with 2-hour rest periods between them [23]. Another study reported unclear to small differences in movement patterns between the first and final matches of a two-day international tournament [21]. On the contrary, state and national-level rugby sevens players demonstrated reduced distances covered at speeds greater than 5 m/s and increased distances covered at lower speeds on the second day of the tournament compared to the first day [24].

Reactive agility is a crucial factor that distinguishes more skilled rugby players from those with lesser abilities [25]. Mastery of reactive agility allows players to quickly adapt to unexpected changes during the game, enhancing their ability to evade opponents and seize scoring opportunities [25]. This study revealed that caffeine supplementation improved reactive agility before the first match on the second day of the tournament. This improvement can give players a competitive edge by enabling better offensive and defensive maneuvers and more effective execution of strategies during matches. Similarly, caffeine ingestion has been shown to improve both reactive agility and decision-making time in team sport athletes [26]. Additionally, a caffeine-based multi-ingredient supplement was effective in enhancing reactive agility in handball players [27].

This study also analyzed the number of collision and repeated high-intensity effort during matches because they are critical indicators of the physical demands unique to rugby sevens. Collisions reflect the contact-intensive nature of rugby, while repeated high-intensity effort encompasses the frequent bursts of high-intensity activities interspersed with brief recovery periods that are central to match performance. These variables directly influence players’ ability to sustain physical engagement and execute tactical roles under fatigue.

The dietary intervention implemented in this study was guided by performance nutrition recommendations for international rugby sevens tournaments [28]. Specifically, a carbohydrate intake of 5–8 g/kg/day is advised on the days leading up to competition to optimize muscle glycogen stores. Additionally, consuming 1–1.5 g/kg of carbohydrates along with 20–25 g of protein within 30 minutes post-match is recommended to maximize muscle repair and glycogen resynthesis [28]. In alignment with these guidelines, our participants consumed approximately 5.5 g/kg of carbohydrates on the day before the trials and 6.2 g/kg on the first day of the trials. Lunch, which provided an average of 1.7 g/kg of carbohydrates, was consumed within 30 minutes following the first match. The protein content of the lunch was kept low due to reports from some participants of stomach discomfort after consuming meals with moderate protein content before exercise. During the trials, the majority of protein intake was allocated to the snack after the second match and to dinner.

The half-life of plasma caffeine concentration after supplementation ranges from 150 to 270 minutes [29]. In this study, there was an interval of approximately 160 minutes between the first and second matches on the same day. To maintain adequate plasma caffeine levels, participants were supplemented with 3 mg/kg of caffeine before each match, resulting in a total daily dosage of 6 mg/kg.

There are several limitations to this study. First, the sample size was small due to the nature of rugby sevens competitions, and an injury to one participant further reduced the sample size. However, this design mitigated the issue of unequal playing time and physiological load between starters and substitutes observed in previous studies [11,20]. Second, while the movement patterns in each match could have been influenced by factors beyond physical capacity, such as tactics, errors, and missed tackles, the use of real matches in this study offers a distinct advantage. This design allows for a comprehensive analysis of the effects of caffeine on various aspects of movement patterns and collisions, which would not be possible with preplanned tasks in controlled environments. Third, the data from participants with different playing positions were pooled together. Although different positions may exhibit distinct movement patterns and physical contacts, it has been shown that physiological and performance characteristics are relatively homogeneous across positions in rugby sevens players [1,30]. Fourth, match strategies of the opposing team and the environmental conditions during the matches were not controlled.

## 5. Conclusion

In conclusion, this study suggested that collegiate male rugby players may increase jogging while reducing walking and standing in the final match of a four-match, two-day tournament following supplementation with 3 mg/kg of caffeine before each match. Additionally, caffeine supplementation enhanced reactive agility before the first match on the second day. This indicates that by alleviating fatigue in the later stages of the tournament, caffeine enabled players to transition from low-intensity activities to higher-intensity efforts. Such adjustments could enhance both offensive and defensive performance during matches. Coaches and sports dietitians may consider incorporating caffeine supplementation in the later stage of 2-day tournaments to provide players with a competitive edge through increased activity levels and better agility. Future studies can consider including participants from multiple teams to increase the sample size and explore the effects of varying caffeine dosages. Investigating rugby-specific skills and decision-making processes, as well as examining plasma and salivary markers for metabolism and recovery, could provide further insights.

## Data Availability

All relevant materials are presented in the present manuscript.

## References

[1] Couderc A, Gabbett TJ, Piscione J, et al. Repeated high-intensity effort activity in international male rugby sevens. J Strength Cond Res. 2023;35(6):1720–13. doi: 10.1519/jsc.0000000000002986 30676389

[2] Suarez-Arrones LJ, Nunez FJ, Portillo J, et al. Running demands and heart rate responses in men rugby sevens. J Strength Cond Res. 2012;26(11):3155–3159. doi: 10.1519/JSC.0b013e318243fff7 22158098

[3] Ball S, Halaki M, Orr R. Movement demands of rugby sevens in men and women: a systematic review and meta-analysis. J Strength Cond Res. 2019;33(12):3475–3490. doi: 10.1519/jsc.0000000000003197 31356510

[4] Henderson MJ, Harries SK, Poulos N, et al. Rugby sevens match demands and measurement of performance: a review. Kinesiology. 2018;50(1):49–59.

[5] Henderson MJ, Fransen J, McGrath JJ, et al. Individual factors affecting rugby sevens match performance. Int J Sports Physiol Perform. 2019;14(5):620–626. doi: 10.1123/ijspp.2018-0133 30427236

[6] Sánchez-Oliver AJ, Domínguez R, López-Tapia P, et al. A survey on dietary supplement consumption in amateur and professional rugby players. Foods. 2020;10(1):7. doi: 10.3390/foods10010007 33375061 PMC7822035

[7] Southward K, Rutherfurd-Markwick KJ, Ali A. The effect of acute caffeine ingestion on endurance performance: a systematic review and meta-analysis. Sports Med. 2018;48(8):1913–1928. doi: 10.1007/s40279-018-0939-8 29876876

[8] Grgic J. Caffeine ingestion enhances Wingate performance: a meta-analysis. Eur J Sport Sci. 2018;18(2):219–225. doi: 10.1080/17461391.2017.1394371 29087785

[9] Grgic J, Trexler ET, Lazinica B, et al. Effects of caffeine intake on muscle strength and power: a systematic review and meta-analysis. J Int Soc Sports Nutr. 2018;15(1):11. doi: 10.1186/s12970-018-0216-0 29527137 PMC5839013

[10] Salinero JJ, Lara B, Del Coso J. Effects of acute ingestion of caffeine on team sports performance: a systematic review and meta-analysis. Res Sports Med. 2019;27(2):238–256. doi: 10.1080/15438627.2018.1552146 30518253

[11] Del Coso J, Portillo J, Munoz G, et al. Caffeine-containing energy drink improves sprint performance during an international rugby sevens competition. Amino Acids. 2013;44(6):1511–1519. doi: 10.1007/s00726-013-1473-5 23462927

[12] Gomez-Bruton A, Marin-Puyalto J, Muñiz-Pardos B, et al. Does acute caffeine supplementation improve physical performance in female team-sport athletes? Evidence from a systematic review and meta-analysis. Nutrients. 2021;13(10):3663. doi: 10.3390/nu13103663 34684665 PMC8538965

[13] Gabbett TJ, Kelly JN, Sheppard JM. Speed, change of direction speed, and reactive agility of rugby league players. J Strength Cond Res. 2008;22(1):174–181. doi: 10.1519/JSC.0b013e31815ef700 18296972

[14] Julien C. What is acceleration (gen2). [cited 2024 Mar 24]. Available from: https://support.catapultsports.com/hc/en-us/articles/360000519736

[15] Julien C. What are repeat high intensity efforts. [cited 2024 Mar 24]. Available from: https://support.catapultsports.com/hc/en-us/articles/360000716535

[16] Cohen J. Statistical power analysis for the behavioral sciences. 2nd ed. (NY) (USA): Routledge Academic; 1988.

[17] Jagim AR, Harty PS, Tinsley GM, et al. International society of sports nutrition position stand: energy drinks and energy shots. J Int Soc Sports Nutr. 2023;20(1):2171314. doi: 10.1080/15502783.2023.2171314 36862943 PMC9987737

[18] Fredholm BB, Battig K, Holmen J, et al. Actions of caffeine in the brain with special reference to factors that contribute to its widespread use. Pharmacol Rev. 1999;51(1):83–133. 10049999

[19] Barcelos RP, Lima FD, Carvalho NR, et al. Caffeine effects on systemic metabolism, oxidative-inflammatory pathways, and exercise performance. Nutr Res. 2020;80:1–17. doi: 10.1016/j.nutres.2020.05.005 32589582

[20] Portillo J, Del Coso J, Abian-Vicen J. Effects of caffeine ingestion on skill performance during an international female rugby sevens competition. J Strength Cond Res. 2017;31(12):3351–3357. doi: 10.1519/jsc.0000000000001763 28002181

[21] Higham DG, Pyne DB, Anson JM, et al. Movement patterns in rugby sevens: effects of tournament level, fatigue and substitute players. J Sci Med Sport. 2012;15(3):277–282. doi: 10.1016/j.jsams.2011.11.256 22188846

[22] Couderc A, Thomas C, Lacome M, et al. Movement patterns and metabolic responses during an international rugby sevens tournament. Int J Sports Physiol Perform. 2017;12(7):901–907. doi: 10.1123/ijspp.2016-0313 27918679

[23] Pereira LA, Nakamura FY, Moraes JE, et al. Movement patterns and muscle damage during simulated rugby sevens matches in national team players. J Strength Cond Res. 2018;32(12):3456–3465. doi: 10.1519/jsc.0000000000001866 28240708

[24] Clarke AC, Anson JM, Pyne DB. Neuromuscular fatigue and muscle damage after a women’s rugby sevens tournament. Int J Sports Physiol Perform. 2015;10(6):808–814. doi: 10.1123/ijspp.2014-0590 25848893

[25] Gabbett TJ, Benton D. Reactive agility of rugby league players. J Sci Med Sport. 2009;12(1):212–214. doi: 10.1016/j.jsams.2007.08.011 18069064

[26] Duvnjak-Zaknich DM, Dawson BT, Wallman KE, et al. Effect of caffeine on reactive agility time when fresh and fatigued. Med Sci Sports Exerc. 2011;43(8):1523–1530. doi: 10.1249/MSS.0b013e31821048ab 21266929

[27] Kaczka P, Maciejczyk M, Batra A, et al. Acute effect of caffeine-based multi-ingredient supplement on reactive agility and jump height in recreational handball players. Nutrients. 2022;14(8):1569. doi: 10.3390/nu14081569 35458131 PMC9025764

[28] Dziedzic CE, Higham DG. Performance nutrition guidelines for international rugby sevens tournaments. Int J Sport Nutr Exerc Metab. 2014;24(3):305–314. doi: 10.1123/ijsnem.2013-0172 24464414

[29] Arnaud MJ. The pharmacology of caffeine. Prog Drug Res. 1987;31:273–313. doi: 10.1007/978-3-0348-9289-6_9 3326033

[30] Fuller CW, Taylor A, Molloy MG. Epidemiological study of injuries in international rugby sevens. Clin J Sport Med. 2010;20(3):179–184. doi: 10.1097/JSM.0b013e3181df1eea 20445357

